# A simulating analysis of the effects of increased joint stiffness on muscle loading in a thumb

**DOI:** 10.1186/1475-925X-8-41

**Published:** 2009-12-16

**Authors:** John Z Wu, Zong-Ming Li, Robert G Cutlip, Kai-Nan An

**Affiliations:** 1 Health Effects Laboratory Division, National Institute for Occupational Safety & Health, Morgantown, WV 26505, USA; 2 Departments of Biomedical Engineering, Physical Medicine and Rehabilitation, and Orthopaedic Surgery, Cleveland Clinic, Cleveland, OH 44195, USA; 3 Division of Orthopedic Research, Mayo Clinic College of Medicine, Rochester, MN 55905, USA; 4 Faculty of Kinesiology, University of Calgary, Calgary, Alberta T2N 1N4, Canada; 5 Department of Mechanical Engineering, West Virginia University, Morgantown, WV 26505, USA

## Abstract

**Background:**

The development of osteoarthritis (OA) in the hand results in increased joint stiffness, which in turn affects the grip strength. The goal of the present study is to theoretically analyze the muscle forces in a thumb in response to the increased joint stiffness.

**Methods:**

The thumb was modeled as a linkage system consisting of a trapezium, a metacarpal bone, a proximal and a distal phalanx. Nine muscles were included in the model: flexor pollicis longus (FPL), extensor pollicis longus (EPL), extensor pollicis brevis (EPB), abductor pollicis longus (APL), flexor pollicis brevis (FPB), abductor pollicis brevis (APB), the transverse head of the adductor pollicis (ADPt), the oblique head of the adductor pollicis (ADPo), and opponens pollicis (OPP). Numerical tests were performed using an inverse dynamic approach. The joints were prescribed to an angular motion at one degree-of-freedom (DOF) each time with all other DOFs of the joints being mechanically constrained, while the muscle forces in response to the joint motions were predicted. The normal joint stiffness was assumed to be 0.05, 0.10, and 0.15 *N m/rad *for interphalangeal (IP), metacarpophalangeal (MCP), and carpometacarpal (CMC) joint, respectively. The joint stiffness was assumed to increase by 50% and 100%, simulating the biomechanical consequences of OA.

**Results:**

Our simulations indicated that the increase in joint stiffness induced substantial increases in muscle forces, especially in the EPL and FPL muscles in response to IP, MCP, or CMC extension/flexion motions.

**Conclusions:**

Because the strength of the muscles in the fingers is limited, the muscles will not be able to overcome joint resistance if joint stiffness is increased to its limit due to OA. This may contribute to the reduced range of motion typically seen in OA.

## Background

The development of osteoarthritis (OA) in the hand is associated with difficulties in gripping activities [1]. Previous studies indicated that the joint stiffness could increase by more than 100% due to OA in the hand [2]. The increased joint stiffness in turn affects the grip strength [3-5]. The biomechanics underlaying the interactions between the muscular loading and joint stiffness variations due to OA has not been investigated. Since it is not convenient to experimentally measure the muscle forces in a finger under physiological conditions, biomechanical models of the hand and fingers are useful for studying such problems.

Multiple biomechanical models of the hands and fingers have been developed to simulate different problems; for example, the whole hand models by [6,7] that simulated the muscle loading for static gripping and free movements, and the biomechanical model of the dynamics of the index finger by [8] that simulated the muscle forces in pinch grip and disc rotation. More practical biomechanical finger models were proposed by [9,10] that included anatomically realistic tendon/muscle network connections in their models. Most of past simulation models and studies were developed for healthy normal hands. The effects of the altered joint stiffness due to pathological conditions, such as OA, on the musculoskeletal loading in a finger has not been analyzed to date.

Direct experimental determinations of the relationship between the muscle force and joint motions in the thumb have been performed by [11,12]. In these experiments, the relationships between the joint motions and muscle forces were tested directly using cadaveric hands. The muscle in the thumb was pulled individually and all other muscles were either loaded by a small force [11] or left free [12], while the joint motions in response to the muscle forces were measured. Again, only healthy donors were considered in these experimental studies.

The goal of the present study is to theoretically analyze the muscle force in a thumb in response to increased joint stiffness. Specifically, we are going to examine: (1) the relationship between the joint motion and muscle force for a normal thumb, and (2) the effects of increased joint stiffness on the relationship between the joint motion and the muscle force. The analysis is to be conducted using an inverse dynamic method, i.e., the joint motion is prescribed while the muscle forces are predicted. We hypothesized that the relationship between the joint motion and muscle forces determined using inverse dynamics will be consistent with those observed in the experimental study [12], and that the elevated joint stiffness will increase the muscle force recruitment.

## Methods

**Figure 1:**
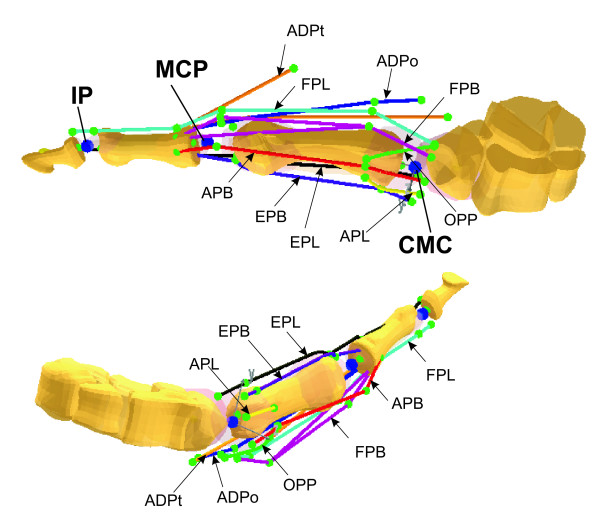
**Schematics of the proposed thumb model developed using AnyBody**. The model consisted of a fixed trapezium, a metacarpal bone, a proximal and distal phalanx, which were linked via three joints: IP, MP, and CMC. Nine muscles were included in the model: FPL, EPL, EPB, APL, FPB, APB, ADPt, ADPo, and OPP.

The thumb was modeled as a linkage system consisting of a trapezium, a metacarpal bone, and proximal and distal phalanges, as illustrated in Figure 1. The trapezium was considered to be fixed. The dimensional scale of the bony sections was consistent with the normative model [13]. These four bony sections were linked via three joints: interphalangeal (IP), metacarpophalangeal (MCP), and carpometacarpal (CMC) joints. The IP joint was modeled as a hinge with one DOF (degree-of-freedom) about the z-axis (extension/flexion), while the MP and CMC joints were modeled as universal joints with two DOFs about the y- (adduction/abduction) and z-axes (extension/flexion). Nine muscles were included in the proposed model (Figure 1): flexor pollicis longus (FPL), extensor pollicis longus (EPL), extensor pollicis brevis (EPB), abductor pollicis longus (APL), flexor pollicis brevis (FPB), abductor pollicis brevis (APB), the transverse head of the adductor pollicis (ADPt), the oblique head of the adductor pollicis (ADPo), and opponens pollicis (OPP). All model parameters in our previous model [14] were adopted in the current study. The terminology describing the muscles as well as the kinematics in the current study are consistent with those in previous studies [15,14]. The tendon attachment locations of each muscle have been calibrated using the experimental data [15] from our previous study [14]. The thumb model was developed on the platform of the commercial software package AnyBody (version 3.0). The bony sections were obtained via CT scanning of one cadaver specimen. The modeling was written in Anyscript code, a program language running on the platform of the AnyBody modeling system. The sign convention was defined consistently for IP, MP, and CMC joints, i.e., extension(-)/flexion(+) and abduction(-)/adduction(+).

The joints were assumed to have a linear stiffness [16]. The joint stiffness was simulated by adding a joint moment () that was proportional to the joint angular () displacement from its neutral position () and in opposite direction to the joint motion:

where  is the added joint moment, *k *is the joint stiffness, and  and  are the current and the neutral joint angles, respectively.

For the MCP and CMC joints, we assumed that the joint stiffness in extension/flexion was identical to that in abduction/adduction motion. The normal joint stiffness, which includes the joint resistance and the effects of the connective tissues, was assumed to be 0.05, 0.10, and 0.15 *N m/rad *for the IP, MCP, and CMC joint, respectively. The joint stiffness was assumed to increase by 50% and 100%, simulating the early stage of OA [2]. The neutral position was considered to be 5 and 10 degrees of the IP and MCP flexion, respectively. All other joint angular components were zero at the neutral position.

Numerical tests were performed using an inverse dynamic approach. The joints were prescribed to an angular motion at one degree-of-freedom (DOF) each time with all other DOFs of the joints being mechanically constrained, while the muscles forces in response to the joint motions were predicted. The joints were moving from their prescribed extreme positions within a time period of 10 s and at constant speeds.

The recruitment of the muscle forces was calculated by using a min/max optimization procedure in AnyBody [17], in which the maximal normalized muscle force was minimized. The minimization of the cost function was subjected to the constraints: the muscle force was greater than or equal to zero and the maximal muscle force did not exceed its capacity, which is estimated by the physiological cross sectional area multiplied by a muscle strength factor of 35 *N/cm*^2 ^[18]. At any instance, the sums of the contributions of each individual muscle to joint moments were calculated and they were balanced with the external forces and the inertial forces of the segments.

## Results

**Figure 2:**
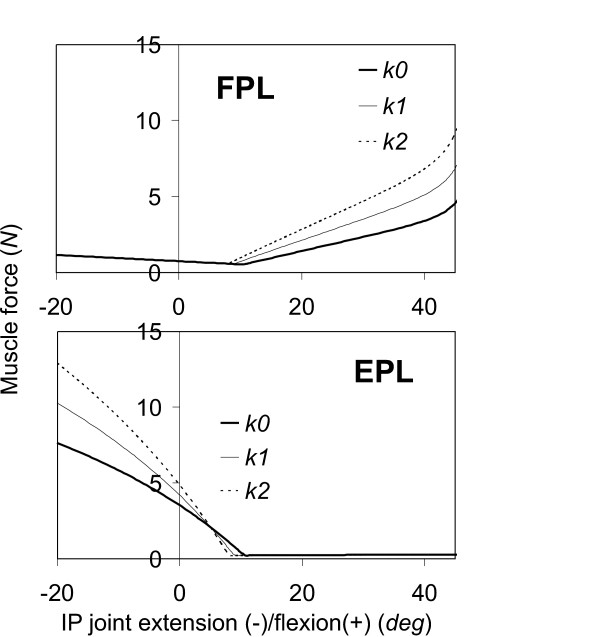
**The predicted EPL and FPL muscle forces as a function of the IP extension(-)/flexion(+)**. The joint stiffness of the thumb was considered to increase by 50% (*k*1) and 100% (*k*2) from the normal thumb (*k*0).

Only two muscles, EPL and FPL, were active in response to the IP extension/flexion; and the forces in all other muscles were negligibly small (Figure 2). The joint stiffness had an obvious effect on EPL and FPL muscles, in which the muscle forces for the joint with increased stiffness were found to increase by approximately 70% and 100%, respectively, compared with those for the normal joint.

**Figure 3:**
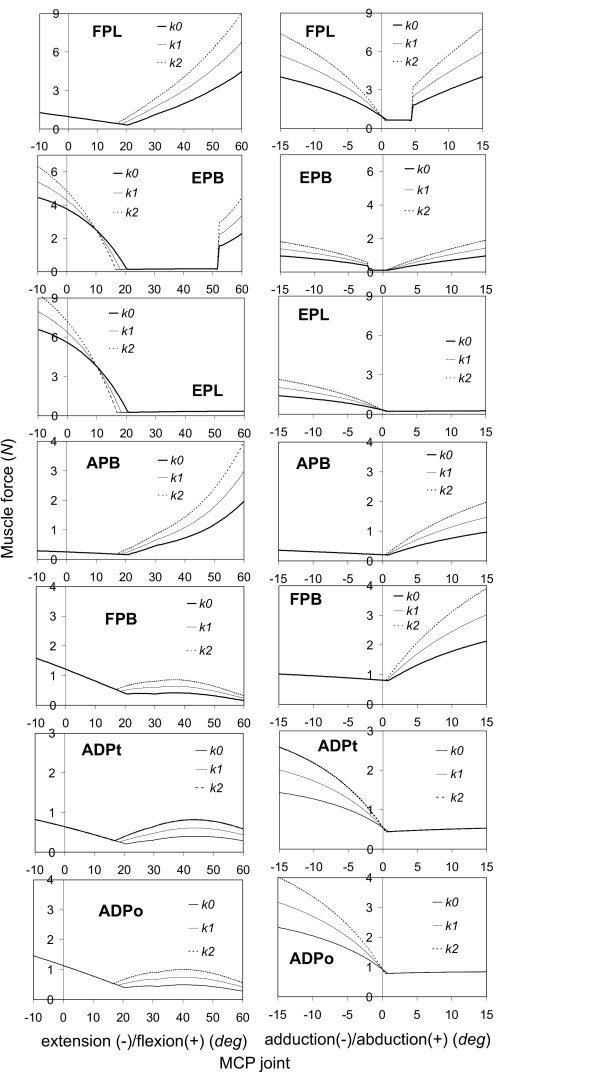
**The predicted forces in the FPL, EPB, EPL, APB, FPB, ADPt, and ADPo muscles as a function of the MCP joint motions**. The left column of the figures shows the muscle force responses as a function of MCP extension(-)/flexion(+), while the right column of the figures shows those as a function of MCP abduction(-)/adduction(+). The joint stiffness of the thumb was considered to increase by 50% (*k*1) and 100% (*k*2) from the normal thumb (*k*0).

**Figure 4:**
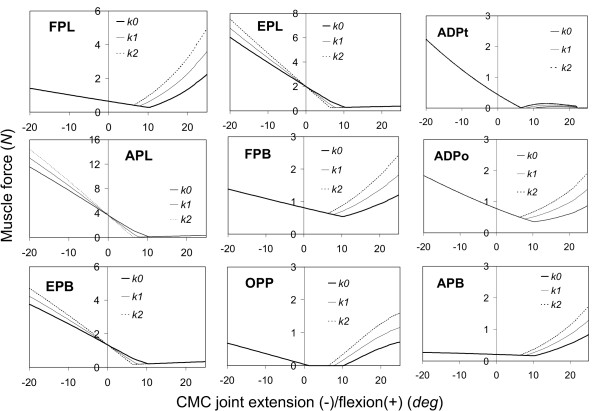
**The predicted forces in the FPL, APL, EPB, EPL, FPB, OPP, ADPt, ADPo, and APB muscles as a function of the CMC extension(-)/flexion(+) motion**. The joint stiffness of the thumb was considered to increase by 50% (*k*1) and 100% (*k*2) from the normal thumb (*k*0).

**Figure 5:**
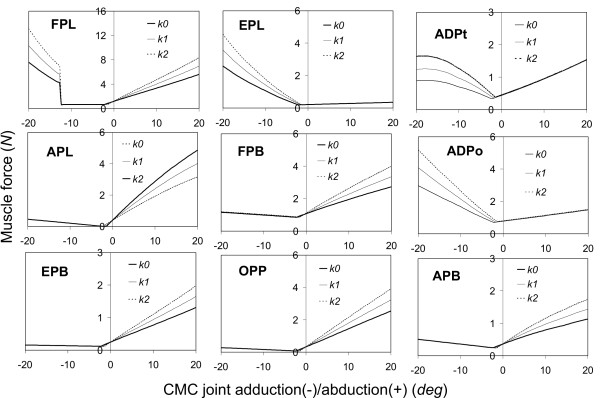
**The predicted forces in the FPL, APL, EPB, EPL, FPB, OPP, ADPt, ADPo, and APB muscles as a function of the CMC abduction(-)/adduction(+) motion**. The joint stiffness of the thumb was considered to increase by 50% (*k*1) and 100% (*k*2) from the normal thumb (*k*0).

The predicted muscle forces in response to the MCP joint motions are shown in Figure 3. The left column of the figure shows the muscle forces corresponding to MCP extension/flexion, while those corresponding to MCP abduction/adduction are shown in the right column. Seven muscles (i.e., FPL, EPB, EPL, FPB, ADPt, ADPo, and APB) were active in response to the MCP joint motions. The muscle forces in APL and OPP were virtually zero and are not shown. The remarkable effect of the joint stiffness on the muscle force in the extension/flexion was observed in the FPL muscle, in which the muscle force was found to increase by approximately 100% due to the increased joint stiffness (*k*2). In the abduction/adduction motion, the greatest effect of the joint stiffness was also found in the FPL muscle, in which the muscle force increased by approximately 87% due to the increased joint stiffness (*k*2). The sudden change of the force in FPL muscle as a function of MCP abduction around zero is due to the contact condition at the muscle/bone surface. The predicted muscle forces in response to the CMC extension/flexion and CMC abduction/adduction motions are shown in Figures 4 and 5, respectively. All nine muscles in the thumb (i.e., FPL, APL, EPB, EPL, FPB, OPP, ADPt, ADPo, and APB) were active during the CMC joint motions. For the extension/flexion motion, the muscle forces in EPL, EPB, and APL were predominant while the greatest effect of the joint stiffness was found in FPL, which showed an increase of approximately 114% muscle force due to the increased joint stiffness. Again, for the abduction/adduction motion, the greatest effect of the joint stiffness was found in FPL muscle force, which showed an increase of approximately 70% due to the increased joint stiffness (*k*2).

## Discussion and Conclusion

Our simulations indicated that the increase in the joint stiffness - a biomechanical consequence in the early stage of OA in the fingers - induces a substantial increase in muscle forces, especially in EPL and FPL muscles in response to the IP, MCP, or CMC extension/flexion motions. Because the strength of the muscles in the fingers is limited, the muscles will not be able to overcome the joint resistance and move the joints through the entire range of motion if the joint stiffness is increased significantly. This explains, in part, why the OA patients suffer a reduced range of motion [19,4].

The predicted relationships between the joint motion and extrinsic muscle (FPL, EPL, APL, and EPB) activities are generally consistent with those observed in the experimental measurements [12]. The relationship between the joint extension/flexion motions and muscle force responses predicted in our simulations agree well with those observed experimentally: the muscle forces in EPL and FPL are generated in response to the IP extension and flexion motion, respectively (Figure 2); in the MCP joint, the muscle forces in EPL and EPB are generated in response to the extension motion while those in the FPL are generated in response to flexion motion (Figure 3-left column); and in CMC joint, the muscle forces in EPL, APL, and EPB are generated in response to extension while that in FPL is generated in response to flexion motion (Figure 4). The relationship between the joint abduction/adduction motions and muscle force responses predicted in our simulations also agree in general with those observed experimentally: the abduction motions in the CMC and MCP joints induce the EPL muscle force (Figures 3-right column and 5); and adduction/abduction motions in CMC and MCP joints induce a small force response in the APL and EPB muscles (Figure 3-right column and Figure 5).

The only difference between the model predictions and the experimental observations [12] is in the FPL muscle in response to the adduction/abduction motions. Our predictions indicated that the MCP and CMC abduction/adduction motions generated force in the FPL (Figure 3-right column and Figure 5), which was, however, not observed experimentally [12]. This does not mean that there is a conflict between the model predictions and the experimental observations. The current simulations indicated that, in order to generate the MCP and CMC abduction/adduction motions, the FPL muscle is required to maintain joint stability in the flexion/extension direction. It should be noted that the current study is different in nature from the experimental study [12]: the simulations were performed using an inverse dynamic technique while the experiments [12] were virtually performed using a forward dynamic principle. In addition, only one muscle was pulled each time and all other muscles had no contributions in the experiments [12], whereas all nine muscles were considered to participate in the force sharing in the simulations.

Another factor of the current study, which could contribute to the difference between the theoretical predictions and experimental data, is the modeling of the MCP and CMC joint. These two joints, especially the CMC joint, are considered to consist of two "scoliotic saddle-shaped" joint surfaces. In the ideal case, there are only two DOFs (extension/flexion and abduction/adduction motions) and the motion along the axis, i.e., pronation/supination, is negligible in such a joint. In a real CMC joint, the pronation/supination motion is not completely constrained because of the flexibility of the cartilage layers and the space in the joint, as demonstrated in the experimental results [12].

In the current study, the joint stiffness is considered as linear and time-independent. Previous experimental data indicated, however, that the relationship of the joint-motion-moment in the fingers is nonlinear [16] and viscous [20]. Because of the effects of the connective tissues, which are nonlinear and viscous, the stiffness property of the joint is also typically nonlinear and viscous. When the joint motion is very slow - the case simulated in the current study - the viscous effects of the joint moment response is negligible. Besides, joint viscosity is conventionally not evaluated in clinical studies. A relationship between the joint viscosity and joint OA conditions has not been established. Therefore, we did not consider the effects of the joint viscosity in the current parametric study.

The curves of the joint moment-angle of the fingers are typically characterized by a hysteresis loop: the joint moment-angle relationship becomes non-linear only towards the ends of the joint motion range, while it is nearly linear around the center of the neutral position. If we fit the joint-motion-moment using a linear model - considering only the joint motion around the neutral position - we need only one parameter (joint stiffness) to describe the characteristics of the joint. However, if the full feature of the joint-motion-moment is modeled using a nonlinear model, we will need more than three parameters. Therefore, the characteristics of the resistance in the joint are conventionally described by using "joint stiffness" in the clinical practice, implying that a linear model is applied to describe the joint-motion-moment around the neutral position. The linear joint stiffness applied in the current study reflects roughly the average joint stiffness as observed experimentally. Technically, it is possible to include the time-dependent and nonlinear characteristics of the joint stiffness into the finger model. However, in order to make it easier for the parametric studies, we felt it was more appropriate to assume a linear joint-motion-moment as in the current parametric study.

In summary, we theoretically analyzed the effects of the increased joint stiffness on muscle loading in a thumb in the current study. Our simulations indicated that the increase in joint stiffness induced substantial increases in muscle forces, especially in EPL and FPL muscles in response to the IP, MCP, or CMC extension/flexion motions. One of the potential applications of the proposed model is the estimation of the joint stiffness inversely using the test data of the gripping tests, which are conducted routinely in clinical diagnostics. The current simulation results might suggest that it is possible, theoretically, to improve the range of motion for OA patients in early stages by increasing muscle strength through exercise.

## Disclaimers

The findings and conclusions in this report are those of the authors and do not necessarily represent the views of the National Institute for Occupational Safety and Health.

## Competing interests

The authors declare that they have no competing interests.

## Authors' contributions

JZW carried out the numerical simulations, participated in the study design, and drafted the manuscript. ZML carried out the study design and helped to draft the manuscript. RGC participated in the study design and manuscript draft. KNA coordinated the study, participated in the study design and manuscript draft. All authors read and approved the final manuscript.
